# Oxidative Stress Control by Apicomplexan Parasites

**DOI:** 10.1155/2015/351289

**Published:** 2015-01-28

**Authors:** Soraya S. Bosch, Thales Kronenberger, Kamila A. Meissner, Flávia M. Zimbres, Dirk Stegehake, Natália M. Izui, Isolmar Schettert, Eva Liebau, Carsten Wrenger

**Affiliations:** ^1^Unit for Drug Discovery, Department of Parasitology, Institute of Biomedical Sciences, University of São Paulo, Avenida Professor Lineu Prestes 1374, 05508-000 São Paulo, SP, Brazil; ^2^Department of Molecular Physiology, Westfälische Wilhelms-University Münster, Schlossplatz 8, 48143 Münster, Germany

## Abstract

Apicomplexan parasites cause infectious diseases that are either a severe public health problem or an economic burden. In this paper we will shed light on how oxidative stress can influence the host-pathogen relationship by focusing on three major diseases: babesiosis, coccidiosis, and toxoplasmosis.

## 1. Apicomplexan Parasites Are Subject to Oxidative Stress from Their Host Cells

Apicomplexan parasites are the causative agents of several different diseases: malaria (*Plasmodium* spp.), toxoplasmosis (*Toxoplasma* spp.), cryptosporidiosis (*Cryptosporidium* spp.), and babesiosis (*Babesia* spp.). Apicomplexa form a large group of complex unicellular eukaryotes and belong to the higher group of the Alveolata along with Chromerida, dinoflagellates, and ciliates. Within the Apicomplexa phylum, all parasites have an infective stage, the sporozoite. The sporozoites enter the host via typical invasion machinery consisting of the apical complex, which is composed of distinct organelles such as rhoptries, micronemes, and dense granules [1]. This process is actin-myosin dependent and subsequently a new host-derived membrane, the parasitophorous vacuole, surrounds the parasite. The life cycles of these parasites are complex, containing asexual and sexual reproduction. However, all these parasites invade cells and have to adapt to the intracellular environment of their hosts. In particular, the apicomplexan parasites have to deal with the oxidative level inside their host cells. Reactive oxygen species (ROS) and oxidative stress are the result of an aerobic metabolism that generates highly reactive metabolites of molecular oxygen (O_2_) in the cytosol or in organelles such as the mitochondria or the peroxisomes [2]. These oxygen metabolites comprise superoxide anions (O_2_^•−^) and hydrogen peroxides (H_2_O_2_) or the highly reactive hydroxyl radical (OH^•^) that is formed in the presence of metal ions via the Fenton and/or Haber-Weiss reactions [3]. Severe discrepancies in the ROS level can induce oxidative modifications in the indispensable cellular macromolecules such as DNA, proteins, and lipids [4], ultimately leading to cell death.

In order to tackle this challenge, parasites have developed a variety of different antioxidant systems such as the thioredoxin- and glutaredoxin-systems. These systems act as thiol/disulfide pairs and are thereby involved in controlling the redox state of the cell. Glutathione/glutathione disulfide (GSH/GSSG) is one of the major redox pairs that control the antioxidative capacity of the cell, while thioredoxins (Trx_red_/Trx_ox_) form an additional redox system that interacts with a different subset of proteins [5]. Trx plays an important role in different biological processes including the reduction of ribonucleotides, transcription control, and hydrogen peroxide detoxification [6, 7]. In addition to these thiol/disulfide pairs, enzymatic antioxidants are also present, which can be classified into primary or secondary antioxidants. Whereas the latter are involved in the regeneration of low molecular weight antioxidant species [8] the primary antioxidants react directly with prooxidants. The enzymes catalase (CAT) and superoxide dismutase (SOD) belong to this class. The CAT catalyses the reaction of 2H_2_O_2_ → 2H_2_O + O_2_ and thereby diminishes the cellular level of hydrogen peroxide. SODs are metallo- (M-) proteins that catalyse the dismutation of superoxide anions to form molecular oxygen and hydrogen peroxide as indicated below (M_ox_ the oxidized and M_red_ the reduced state of the metalloproteins):

(1) Mox+O2•−  ⟶Mred+O2Mred+O2•−  +2H+⟶Mox+H2O2

Several common forms of SODs exist that are classified according to their metal cofactors such as Cu/Zn, Fe, or Mn. Different SODs can be present in a single cell; for example, the mammalian cells contain cytosolic Cu/ZnSODs and MnSODs in their mitochondria [9].

Antioxidants also include peroxiredoxins (Prx) which are involved in the conversion of peroxides and alkyl hydroperoxides to water or the respective alcohol and have two characteristic catalytic cysteine residues [10]. Furthermore, glutaredoxins utilize the reducing power of glutathione to catalyze disulfide reductions in the presence of NADPH and glutathione reductase (the glutaredoxin system).

Due to the fact that the redox systems play such a fundamental role for parasites [3] this paper highlights the importance of oxidative stress for host-pathogen interactions in apicomplexa; however, due to the recent article by Nepveu and Turrini [11] we would like to focus on different apicomplexan parasites other than* Plasmodium*.

## 2. *Babesia*: Infection and Host Response

Parasites of the genus* Babesia* are classified as apicomplexan and belong to the suborder* Piroplasmida* within the family* Babesiidae*. This classification is based on their capacity for invading erythrocytes, multiplication via budding rather than schizogony, and the lack of hemozoin [12].* Babesia gibsoni* is a pathogen occurring in Indian dogs. The first species was described by Patton in 1910, and since then the disease has been widely reported all over the world [13–15].

*Babesia* spp. are naturally transmitted by the bite of infected ticks, from the species* I. ricinus*. Other occasionally occurring mechanisms of transmission are via transplacental and perinatal routes and from contaminated blood products [15]. Among the known species,* B. microti*,* B. divergens*, and* B. venatorum* cause human babesiosis in Europe [12, 16–18].

Similar to other members of this group,* Babesia* undergoes a complex life cycle involving arthropods and other mammalian hosts [19]. The cycle starts with ticks taking a blood meal thereby infecting the mammalian host with sporozoites [20], which then invade erythrocytes and reproduce through asynchronous binary fission. This results in two or sometimes four merozoites. Once present in a reservoir host,* Babesia* will develop into male and female gametes [18, 21].

The zoonotic* Babesia* reservoirs are quite diverse species including the white-footed mouse, cattle, wild ruminants, canids, shrews, and possibly cottontail rabbits. Several reservoirs are unknown for some human* Babesia* pathogens [18]. The cycle is completed when an ixodid tick feeds on a competent reservoir and the gametes fuse to form the zygote. Finally the pathogen undergoes a sporogonic cycle, forming the infectious sporozoites [20, 22].

The members of* Babesia sensu stricto* spp. group have a characteristic feature in common; they can infect ovary cells and thus be transmitted transovarially by eggs [12]. Despite this information, there are several species of* Babesia*, such as* B. duncani*, which are almost not characterised [16].

Like many aerobic parasites,* Babesia* lives in an oxygen-rich environment within its mammalian host (mainly during the erythrocytic stage). As a result, the pathogen is exposed to the toxic effects of ROS, which can cause damage to membrane lipids, nucleic acids, and proteins [23, 24].* Babesia* is an example of the importance of the parasite's antioxidant system for proliferation within erythrocytes [25–27].

According to Regner et al., the biochemical properties of proteins of the Trx system from the bovine parasite* B. bovis*, the* Bbo*Trx(R) system, share several features with their counterparts in* P. falciparum*, such as kinetics and physical properties and the capacity for reducing* S*-nitrosoglutathione (GSNO) and GSSG [26] (Table 1).

This latter competency is highly relevant, since GSH is the most abundant intracellular nonprotein thiol that represents a key-molecule within redox homeostasis. To date, three genomes of the genus* Babesia* were sequenced:* B. bovis*,* B. microti*, and* B. equi*. After sequence analysis of the* B. bovis* genome database, no homology to a specific glutathione reductase was identified, suggesting that* B. bovis* might lack this enzyme. Further, the 2-Cys peroxiredoxin present in the cytoplasm of the merozoite was characterized to be an important component of the Trx network in* B. bovis* and* B. gibsoni*, which is able to reduce ROS [23, 28].

In general,* Babesia* spp. parasites, as well as species of* Theileria* and* Plasmodium*, invade erythrocytes and cause anaemia of the host [18, 29, 30]. Anaemia is considered as a severe complication of babesiosis, being the major cause of mortality in infected animals. However, its pathogenesis still remains uncertain [18]. Despite the correlation between parasitism and anaemia, the severity of anaemia is not always proportional to the parasitaemia [31, 32]. This phenomenon suggests that nonparasitized erythrocytes may also be damaged by an unknown mechanism of action [32].

The parasite does not always need to control elevated ROS levels present in the host. Studies in dogs naturally infected with* B. gibsoni* demonstrated the presence of a host response via an increased expression level of the SOD and CAT enzymes implying the generation of ROS by the pathogen. Additionally, an elevated level of lipid peroxides within the erythrocyte was detected. Moreover, in this study it was suggested that the low level of iron, zinc, and copper in the blood seems to have an additional role in the genesis of anaemia and oxidative stress [32].

In parasites that are proliferating in erythrocytes, the liver plays an essential role in clearing infected red blood cells [30]. It has been shown by flow cytometry analysis that* B. divergens* induces hepatic tissue damage via oxidative stress, leading to an alteration in the cell metabolism. Further, it has been demonstrated that ROS damage hepatocytes, thereby affecting the function of the liver [33]. This study also observed a significant decrease in the total antioxidant capacity during* B. divergens* infections. This was shown by decreased GSH and CAT levels as well as a significant increase in the concentration of malondialdehyde (MDA). The increased level of MDA strongly suggests lipid peroxidation and alteration of the nitrite/nitrate levels [34]. Increased levels of MDA have also been reported in* B. gibsoni* infections [32] and in double infections of* Ehrlichia canis* and* B. gibsoni* [34, 35].

Currently there are several drugs used for the treatment of human babesiosis. Atovaquone, azithromycin, clindamycin, and quinine are drugs that show activity in* Babesia* animal models [12]. Due to spreading drug resistance, there are currently only two major antimicrobial treatments present, which consist of a combination therapy of antimalarial drugs and antibiotics such as quinine and clindamycin or atovaquone and azithromycin [12, 36, 37]. The use of antibiotics is considered to target the apicoplast, an organelle which is also present in the apicomplexan parasites* Toxoplasma* and* Plasmodium* [38].

## 3. *Cryptosporidium*: A Resistant Pathogen

The protozoan pathogen* Cryptosporidium* was described first by Tyzzer, which is a worldwide occurring coccidian parasite causing gastroenteritis in mammals. The main pathway of infection is the uptake of oocysts via food consumption [39, 40].

*Cryptosporidium *spp., in contrast to other apicomplexan parasites, have a monoxenous life cycle that takes place in the gastrointestinal tract of the host. During the excystation, four infective sporozoites, which are released from the oocyst, glide over the intestinal cells until they invade the cell using the apical complex. After infection the parasite develops at the apical surface in the host cell; thus the parasite is in an intracellular but extracytoplasmic state. Inside the parasitophorous vacuole,* Cryptosporidium* spp. are protected from the gut environment and use nutrients via an Apicomplexa-unique feeder organelle. Uniquely amongst other apicomplexan parasites,* Cryptosporidium* spp. lack an apicoplast and also have lost the mitochondrial genome and most of its functions [41, 42].

The first report of cryptosporidiosis was in the early 20th century while the first case in humans was reported in 1976 [43–45]. Today* Cryptosporidium* spp. are known as the major waterborne parasite worldwide with an important economic impact and a source of diarrhea in calves and lambs [46]. Although* Cryptosporidium* spp. are also pathogenic for humans, clinical symptoms such as diarrhea are restricted to immune-deficient people. However, immune-compromised patients (e.g., HIV/AIDS patients) are at higher risk and cryptosporidiosis can lead to dehydration, wasting, and even death [47–54].

While various* Cryptosporidium* spp. were detected in humans, over 90% belong to the most common species* C. hominis* and* C. parvum* [55, 56]. Little is known about their pathogenic factors due to difficulties in* in vitro* culturing and only oocysts have been biochemically analyzed so far [57–59]. To date, the genome sequencing project of* C. hominis* and* C. parvum* has discovered about 25 putative virulence factors [58].

One of these factors is an acid phosphatase in* C. parvum* [60]. Aguirre-García and Okhuysen have shown the activity of the membrane-bound enzyme in the oocyst [60]. They suggest that the acid phosphatase similar to those in* Leishmania* spp.* Coxiella burnetii*,* Legionella micdadei*, and* Francisella tularensis* has an important role for the survival of the intracellular pathogens [61–64]. Acid phosphatases are known to inhibit the respiratory burst of human neutrophils and macrophages [65–67].

Only limited information is present of the antioxidant enzymes in* Cryptosporidium* spp. While the enzymes glutathione transferase, glutathione reductase, and glutathione peroxidase seem to be absent in* C. parvum* (Table 1), some SOD activity has been detected [68]. Further, it has been shown that the parasite contains and synthesizes GSH and possesses a thioredoxin peroxidase that could be important for detoxification and thereby protection against ROS [69–72] (Table 1). Previous studies suggested that the antioxidant enzymes might have a protective effect against ROS that occurs via inflammation or phagocytosis by macrophages [68].

To enhance the negative effect of oxidative stress on* C. parvum*, the use of selenium (Se) seems promising [73, 74]. Selenium compounds are able to react with thiols such as GSH which lead to elevated levels of superoxide and hydrogen peroxide [75]. Interestingly, Se-supplemented* C. parvum*-infected mice show a decreased number of oocysts in feces and a longer survival time than the respective control [73]. On the other hand Se-deficiency results in a heavy oocyst shedding, a higher susceptibility of the host to* C. parvum*, and a decreased immune response [73, 74].

A major problem is the high resistance of the parasite to common disinfectants such as chlorine [48, 76, 77]. The disinfecting effect of chlorine is based on free radicals, which seem to react with the plasma membrane [78]. As a response* C. parvum* initiates the expression of the chaperone Hsp70 [79]. Bajszár and Dekonenko suggest that this chaperone protects membrane proteins against protein denaturation caused by oxidative stress, as already known for the bacterial Hsp33 [79, 80].

## 4. *Eimeria*: Effect of Diet Supplementation upon Oxidative Balance

*Eimeria* spp. are etiologic agents of coccidiosis [81]. Although some organisms such as reptiles, mammals, and fish can be infected with coccidia, the majority of the studies were focussed on species infecting poultry, such as* E. tenella*,* E. brunetti*,* E. praecox*,* E. mitis*,* E. acervulina*,* E. necatrix*, and* E. maxima*. Due to the huge economic significance, there is an urgent need for strategies to control the parasite [82, 83].* Eimeria* ssp. usually differ in pathogenesis and tissue tropism. The transmission of coccidiosis is facilitated by a faecal-oral-mechanism of contaminated water or food which initiates the comprehensive life cycle [83].

The current strategy for coccidiosis control relies on (i) the parasite's cofactor metabolism (synthetic drugs), such as ethopabate, sulphonamides, pyrimethamine, and amprolium [84], (ii) the mitochondrial metabolism, employing drugs such as quinolone which blocks electron transport [85] and the triazinetrione compound toltrazuril, which reduces the activity of certain enzymes in the respiratory chain [86]; and (iii) the balance of ions, using drugs like polyether antibiotics or ionophores which induce osmotic damage [87]. Furthermore, some drug combinations of currently unknown mode of action have shown promising effects [88]. However, as already known from other drug based therapies, the indiscriminate use of anticoccidian compounds leads to the development of resistant strains [89].

*Eimeria* infections can harm the host, among other classically described mechanisms [22], due to an imbalance of the antioxidant defence system [90, 91]. In order to control the oxidative environment of the host,* E. tenella* increases the level of antioxidative enzymes such as CAT during infection [90].

As outlined above, interfering with the redox homeostasis makes the cell vulnerable to ROS and can cause cell damage. Classical biological markers such as MDA and lipid peroxidation (LPO) have elevated concentrations in* E. tenella*- and* E. acervulina*-infected birds [92–94].

Sepp and colleagues showed that greenfinches fed on an excess of carotenoids as an antioxidant were able to manage chronic* Eimeria* infection [95]. Based on this it has been suggested to use food supplements such as 2Gly·ZnCl_2_·2H_2_O [92], vitamin A, vitamin E, vitamin C, and low-molecular endogenic antioxidants for controlling* E. tenella* infections [82, 96, 97].

## 5. *Toxoplasma*: Oxidative Stress, a Source of Drug Targets

Infection with the parasite* Toxoplasma gondii* has a worldwide distribution [22]. The parasite is transmitted by warm-blooded animals, from the mother to fetus (congenital) and also by food-borne transmission [22]. In many cases the immune system can prevent the symptoms; however,* T. gondii* infections can be lethal for immune deficient people such as HIV patients [98]. Moreover, during pregnancy the fetus is at particular risk since the disease can affect the nervous system, eyes, and other organs [99].

The life cycle of* T. gondii* begins with the ingestion of tissue cysts by cats, the definitive host. Once inside the (human) intestine, the oocyst excysts and subsequently the tachyzoites invade cells using a characteristic active invasion mechanism. Within the tissues* T. gondii* is infective for various cell types. Like the malaria parasite* T. gondii* forms a parasitophorous vacuole during the invasion process [100].

In the parasitophorous vacuole the parasite already induces the production of IL-10 (interleukin 10) and TGF-*β* (transforming growth factor *β*). In this manner,* T. gondii* is able to modulate the innate and adaptative host immune system in order to reduce the immune response rather than its complete inhibition [101]. The stress deriving from the host's immune system can induce differentiation from the highly replicative and invasive form (tachyzoite) towards the persisting bradyzoite stage [102].

In the early phases of invasion, macrophages and natural-killer cells are primarily responsible for defeating the parasite [103]. Classically, these cells use ROS against pathogens; therefore the antioxidant defence system of* T. gondii* became attractive for drug discovery.

**Figure 1:**
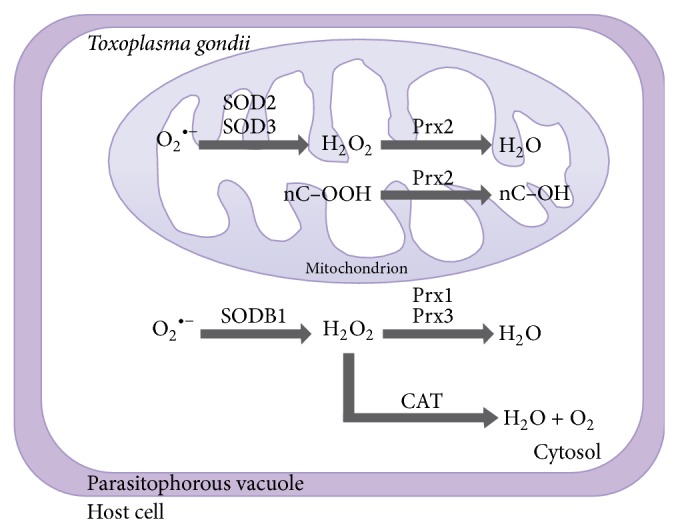
*Toxoplasma gondii* peroxidases: the cytosolic superoxide dismutase (SODB1) is involved in the conversion of superoxide anion (O_2_^•−^) into hydrogen peroxide (H_2_O_2_) followed by either the conversion to water by the peroxiredoxins (Prx1 and Prx3) or the conversion to water and molecular oxygen (O_2_) by the enzyme catalase (CAT). Within the mitochondrion, the superoxide anion can be converted to hydrogen peroxide via two different superoxide dismutases (SOD2 and SOD3), which can be converted to water when coupled with the 1-cys peroxiredoxin. Prx2 is also involved in the detoxification of organic peroxides (for instance, the nC-OOH) towards the respective alcohol [102, 104, 107, 109, 121].

Regardless of the CAT's importance in detoxifying H_2_O_2_, this enzyme is lacking in most pathogenic protozoans [8]. Unusually, the* T. gondii* genome encodes for a catalase [104]. Based on similarity analysis with respective homologues, the enzyme requires NADPH and a haem group as cofactors [105]. Much had been discussed about the localisation and role of the catalase in the parasite. Initially a classical peroxisomal localisation was suggested, whereupon the parasite would use the catalase for detoxifying by-products within peroxisomes [10]. However, the cytosolic localisation has been recently confirmed by microscopy and corroborated by the absence of peroxisome biogenesis factor (PEX) proteins in* T. gondii* (Figure 1) [106]. A cytosolic catalase can act on the detoxification of the majority of host born peroxides due to its high substrate turnover [104, 105].

Additionally, catalase seems to have an important role in invasion and replication inside the parasitophorous vacuoles according to knock-out studies [107]. Here, the knock-out cell line was more susceptible to peroxide exposure and was also less virulent to mice [107]. This is consistent with studies demonstrating that reduced catalase expression could diminish the infection efficacy in mice [108].

Peroxiredoxins can support the catalase in its detoxification of reactive oxygen species. Currently, three peroxiredoxins have been found in* T. gondii*. They have different catalytic mechanisms, subcellular localisations, and roles in the parasite metabolism.

The peroxiredoxin 2 (Prx2, Figure 1) belongs to the 1-cys group which, unlike the other peroxiredoxin groups, possesses just one catalytic cysteine residue. Despite this dissimilarity, Prx2 is still able to detoxify H_2_O_2_ in the presence of dithiothreitol [107, 109]. It is worth mentioning that up to date no endogenous reducing partner has been identified, despite the fact that glutathione, lipoic acid, thioredoxin, and glutaredoxin have all been tested as electron donors for the regeneration of the protein [109]. Prx2-overexpressing parasites showed an increased resistance against H_2_O_2_ stress, which allowed the pathogen to survive after applying oxidative stress [107]. Additionally, further experiments suggest another role for Prx2. The 1-cys peroxiredoxins are bifunctional enzymes with phospholipase activity and its overexpression is related to membrane protection against oxidative stress [110, 111].

Furthermore,* T. gondii* possesses two other peroxiredoxins, Prx1 and Prx3, which belong to the typical 2-cys peroxiredoxins habouring two cysteine residues for forming intermolecular disulphide bonds [10, 112]. Normally peroxiredoxins are considered as proteins with a slow conversion rate [10]. The* Tg*Prx1 is a cytosolic enzyme with constitutive expression which, differentially from Prx2 and Prx3, has an unusually high efficiency in dealing with peroxides (Figure 1, Table 2) [109].

Prx3 is localised in the mitochondrion and there likely to be involved in the detoxification of its own metabolism-derived ROS and their respective by-products [107, 113, 114]. The function of Prx3 might be assisted by another set of enzymes; two SODs are also present in the parasite's mitochondrion (Table 2) [107].

SOD2 and SOD3 have been localised in the parasite's mitochondrion. They have conserved residues to bind iron and although very similar in the primary sequence to SODs from* P. falciparum*, their configuration is more characteristic to prokaryotes [107]. There is a third cytosolic superoxide dismutase, SODB1, previously characterized as Fe-binding enzymes, which is different to the Mn-binding enzyme in humans. Studies have demonstrated that this gene is essential, as SODB1 knock-outs are lethal [115]. SODs are present in almost all developmental stages of* T. gondii* and in all organelles, pointing out the importance of a rapid detoxification of superoxide anions in order to protect the parasite.

The classical antioxidant systems such as Trx and GSH are also suggested to occur in* Toxoplasma*, which is corroborated by the presence of* trx* genes in the transcriptome as reviewed previously [116]. The glutathione biosynthetic enzymes are present in the genome of* T. gondii* (Table 2). However, no experimental data are available about these enzymes.

Currently, the treatment of toxoplasmosis relies on inhibitors such as pyrimethamine and atovaquone, in a similar way to the treatment of malaria [117]. In malaria artemisinin has been proposed to interfere with the parasite's oxidative homeostasis, today being one of the most effective drugs for malaria treatment. Artemisinin has also been experimentally used against* T. gondii*, however, with only moderate effect in comparison to malaria [118, 119]. This, besides other factors, might underline the importance of the antioxidant systems for the parasite [120].

Regardless of the variety of antioxidant systems presented in this review,* T. gondii* is still highly susceptible to oxidative stress as demonstrated by the use of stress inductors, such as juglone, phenazine methylsulfate and* t*-BuOOH, affecting the tachyzoites stage even at the nanomolar range, without harming the host [109]. The antioxidant systems in* T. gondii* are essential and therefore good candidates for the discovery of novel drugs.

## 6. Conclusion

This review highlights the importance of the antioxidant systems of apicomplexan parasites, which are essential for tackling oxidative stress and consequently indispensable for the survival of the pathogens in their hosts. Parasites such as* T. gondii* and* Babesia* have developed a complex antioxidant defence system for surviving inside the host cell. Oxidative stress not only is a problem for intracellular survival but also occurs during infection when the parasite is exposed to the host's immune system, which uses ROS to fight the infection. In summary all apicomplexa are highly susceptible to oxidative stress and therefore selective interference with the redox homeostasis of these pathogens presents excellent drug target qualities.

## Figures and Tables

**Table 1:** Predicted antioxidant enzymes according to the genome databases of *Babesia* and *Cryptosporidium*.

Proteins	Abbr.	*B. bovis *	*B. equi *	*C. hominis *	*C. parvum *	References
Directly dealing with prooxidants						
Glucose-6-phosphate dehydrogenase		BBOV_IV001600	BEWA_012440A			[122, 123]
Superoxide dismutase (SOD1) Fe	SODB1	U70131	BEWA_043090	XM_660499	AY599065	[124, 125]
Peroxiredoxin	Prx1	—	BEWA_033010	—	—	[23, 28]
Glutathione system						
Glutathione peroxidase 1		—	—	XM_663205.1	XM_626631.1	[58, 68, 126]
Glutathione reductase						[68]
Glutathione synthase		XP_001610411	XM_004829930	—	—	[122, 123]
Glutaredoxin	Grx	BBOV_IV00432023	BEWA_031250	XM_660840	XM_627733	[58, 122, 123, 126]
Thioredoxin system						
Thioredoxin	Trx	BBOV_II003650	BEWA_047060	—	XP_626144	[26, 126]
Thioredoxin peroxidase 1		AK440717	BEWA_001220	XM_660495	GQ388272	[72]
Thioredoxin reductase	TrxR	BBOV_I002190	XM_004831637	GQ388271	AY145120	[26]

**Table 2:** Antioxidant enzymes in *T. gondii*.

Proteins	Abbr.	*T. gondii *	Comments	Stage	Localisation	References
Directly dealing with prooxidants						
Catalase	CAT	AF161267		Constitutive	Cytoplasmatic	[104, 105, 107]
Glucose-6-phosphate dehydrogenase		XP_002370586	Putative			
Superoxide dismutase (SOD1) Fe	SODB1	AF029915	Fe in the active site	Tachyzoite/bradyzoite	Cytoplasmatic	[115]
Superoxide dismutase (SOD2)	SOD2	AY176062		Constitutive	Mitochondrial	[107]
Superoxide dismutase (SOD3)	SOD3	AY254045		Sporulated oocyst	Mitochondrial	[107]
Peroxiredoxin	Prx1	AF305718	2-Cys mechanism	Constitutive	Cytoplasmatic	[107, 127]
Peroxiredoxin	Prx2	AF397213	1-Cys mechanism	Tachyzoite/bradyzoite	Cytoplasmatic	[107]
Peroxiredoxin	Prx3	AY251021	2-Cys mechanism	Constitutive	Mitochondrial	[107]
Glutathione system						
Glutathione peroxidase 1		AY043228	Putative	Tachyzoite/bradyzoite		
Glutathione reductase		AF041450	Putative			
Glutathione synthase		XP_002366333	Putative			
Glutathione-S-transferase		XP_002369230	Putative			
*γ*-Glu-Cys synthase		XP_002368128	Putative			
Glutaredoxin	Grx1	BM131493	Putative			
Glutaredoxin	Grx2	BM040167	Putative			
Thioredoxin system						
Thioredoxin	Trx	BG657266	Putative	Constitutive		[117]
Thioredoxin peroxidase 1		BM039715	Putative	Oocyst		[117]
Thioredoxin reductase	TrxR	AA519618	Putative			
